# Sevoflurane Induction With Snorkel Technique in an Adult With Mask and Needle Phobia

**DOI:** 10.7759/cureus.58777

**Published:** 2024-04-22

**Authors:** Balaji Vaithialingam, Sujith Raju

**Affiliations:** 1 Division of Neuroanaesthesiology, International Institute of Neurosciences, Aster Whitefield Hospital, Bengaluru, IND

**Keywords:** face mask, anesthesia, needle phobia, sevoflurane induction, snorkel breathing

## Abstract

Snorkel breathing involves mouth breathing into a snorkel tube and is necessary for underwater activities. Anesthesiologists may have difficulty when dealing with adults who have a concomitant face mask and needle fear because both inhalation and intravenous induction cannot be performed. This case report describes a novel use of the snorkel breathing technique for anesthetic gas induction in an adult with face mask fear. A 24-year-old female with mask and needle fear underwent a craniotomy and biopsy of a frontal lesion while under general endotracheal anesthesia. During anesthesia induction, the patient was directed to hold the breathing tube tightly between her lips and breathe via her mouth into it with sevoflurane at 8% dial setting and 6 L/min of fresh gas flow. The snorkel approach was effectively used to induce anesthesia with better patient cooperation, and an intravenous cannula was inserted.

## Introduction

Sevoflurane gas induction is the conventional approach for anesthetic induction in pediatric patients. It also serves multiple functions, including difficult airway management, rapid sequence anesthetic induction, and day-care anesthesia administration [1]. Inhalational induction has recently emerged as a safer anesthetic technique for elderly people and patients with limited cardiovascular reserve [2,3]. Adults are substantially more cooperative than children; thus, inhalation induction is much smoother and easier. However, anesthesiologists may encounter difficulty when dealing with very phobic adults requiring inhalational induction because face mask application is not always feasible. We propose a unique use of the snorkel breathing technique for sevoflurane gas induction in a very anxious adult with a concomitant face mask and needle phobia.

## Case presentation

A 24-year-old female with no medical history presented with a mild, generalized headache. The magnetic resonance imaging of the brain revealed a right frontal lesion, and a biopsy of the lesion was planned under general endotracheal anesthesia. During the pre-anesthetic check-up, the patient expressed concern about anesthesia because she had never been exposed to it previously. The patient also exhibited significant apprehension over the face mask application and insisted on needle placement under anesthesia. Given her dislike of masks, the patient was advised to undergo an inhalational induction utilizing a snorkel technique, and anesthetic consent was obtained.

**Figure 1:**
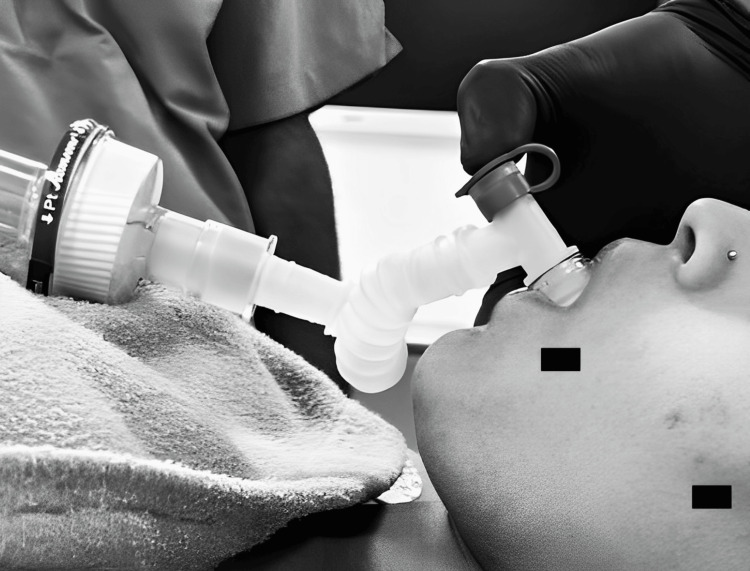
Conduct of sevoflurane induction with snorkel breathing in a phobic patient

A five-lead electrocardiography (ECG), non-invasive blood pressure (NIBP) monitor, and a peripheral pulse-oximeter (SPO_2_) were attached, and baseline vital signs were recorded in the operating room with a female attendant present. The patient had a baseline heart rate of 130 bpm and a blood pressure of 130/80 mmHg. The attending anesthesiologist demonstrated the snorkel breathing technique with the respiratory circuit to the patient before anesthesia induction. The circle system, which included a reservoir bag, was primed and pressurized with sevoflurane (8% dial setting) at an oxygen-fresh gas flow of 6 L/min for three minutes. The patient was instructed to hold the anesthesia breathing tube snugly between her lips and inhale/exhale through her mouth into the breathing tube (with an open adjustable pressure limit valve) at an initial sevoflurane dial setting of 8% and fresh gas flow (100% oxygen) of 6 L/min (Figure 1). 

After 45 seconds of normal tidal breathing with the snorkel technique and at a minimal alveolar concentration (MAC) of 0.6-0.7, there was no response to verbal commands, and involuntary movements began. The snorkel breathing technique was replaced with standard face mask breathing, and the sevoflurane dial setting was reduced to 3%. A 20-G intravenous cannula was secured upon reaching 1.1 MAC with the patient breathing spontaneously, and the trachea was intubated after being paralyzed with cis-atracurium (0.2 mg/kg).

## Discussion

Snorkel breathing is essential for underwater activities because it allows a surface swimmer to observe the underwater world through a face mask without being disturbed by inhalation [4]. It consists of inhalation and exhalation through the mouth into a snorkel tube with a closed nose. Sleth et al. discussed the benefits of the snorkel breathing technique for pre-oxygenation during anesthesia induction [5]. The snorkel breathing approach eliminates the need for a face mask and reduces plastic waste in the operating room. Apneic oxygenation can also be achieved via the snorkel technique, which involves keeping the lips closed around the breathing tube hose. We used the snorkel breathing method in a completely different context. Pre-oxygenation using a face mask is a routine procedure before anesthetic induction, and it is required to sustain apnea during tracheal intubation.

There are several strategies for overcoming mask phobia in pediatric patients, including the use of a scented mask and anesthetic induction with parental supervision [6-8]. However, no such techniques have been identified for adults experiencing mask fear. Anesthesiologists may have difficulty when dealing with adults who have both mask and needle fear because inhalation and intravenous induction cannot be performed. Forceful needle insertion or face-mask application can result in vasovagal syndrome and patient displeasure. Our patient tolerated the snorkel breathing without hesitation, which aided in a smooth inhalation induction with sevoflurane and the placement of an intravenous line. The sevoflurane induction with the snorkel approach can be done with a single vital capacity breath or with tidal volume breathing.

To expedite the process, anesthetic gases should be primed and pressurized into the respiratory circuit. Active mouth blowing during snorkel breathing is only achievable in awake and cooperative patients, so we switched to a standard face mask when there was no response to verbal commands at 0.6 MAC.

## Conclusions

Anesthetizing an adult patient with mask and needle phobia can be difficult. This report addresses an essential practical issue that anesthesiologists may face while dealing with an adult with extreme phobia. Inhalation induction with snorkel breathing may be an option for starting a smooth anesthetic induction in a patient who is resistant to facemask application and has a needle fear. Snorkel breathing is simple to execute and is more acceptable in adult patients during anesthesia induction because it does not require the use of a face mask.
